# Comparative quantitation of liver-type fatty acid-binding protein localizations in liver injury and non-pathological liver tissue in dogs

**DOI:** 10.14202/vetworld.2024.313-318

**Published:** 2024-02-07

**Authors:** Jirapat Arunorat, Nuttawan Chusakulwong, Natcha Sakunasing, Pitchaya Matchimakul

**Affiliations:** 1 Department of Veterinary Biosciences and Veterinary Public Health, Faculty of Veterinary Medicine, Chiang Mai University, Chiang Mai 50100, Thailand; 2 Research Center for Veterinary Biosciences and Veterinary Public Health, Faculty of Veterinary Medicine, Chiang Mai University, Chiang Mai 50200, Thailand; 3 Academic Year 2565, Department of Veterinary Biosciences and Veterinary Public Health, Faculty of Veterinary Medicine, Chiang Mai University, Chiang Mai, 50100, Thailand

**Keywords:** canine, hepatocyte, immunohistochemistry, lipidosis, liver injury, liver-fatty acid-binding protein, pathology

## Abstract

**Background and Aim:**

Liver injury results in the production of free radicals that can lead to hepatocytic degeneration, cirrhosis, and hepatocellular carcinoma (HCC). Liver-fatty acid-binding protein (L-FABP) is highly expressed in hepatocytes and is a key regulator of hepatic lipid metabolism and antioxidant characteristics. Interestingly, the increase in L-FABP expression could be used as a novel marker of liver injury. Therefore, this study aimed to use immunohistochemical techniques to investigate the expression of L-FABP in dogs with liver injury compared with dogs with non-pathological liver.

**Materials and Methods:**

Liver tissue samples were collected from dog biopsy specimens at the Veterinary Diagnostic Laboratory at the Faculty of Veterinary Medicine, Chiang Mai University. The tissues were prepared for immunohistochemistry and the expression and localization of L-FABP were investigated using one-way analysis of variance.

**Results:**

Immunohistochemical analysis showed that L-FABP was strongly expressed in the hepatocytes of dogs with lipidosis and HCC when compared with that in normal liver. Semi-quantitative immunohistochemistry evaluation showed the percentage of protein expression of L-FABP 0.023 ± 0.027 in the non-pathological liver. The percentage of L-FABP protein expression in lipidosis and HCC was found to be 8.517 ± 1.059 and 17.371 ± 4.026, respectively.

**Conclusion:**

L-FABP expression in dogs with liver injuries was significantly higher than that in dogs with non-pathological liver injury (p = 0.05). These results suggest that L-FABP has the potential as a novel marker for specific diagnosis and prognosis of dogs with liver injury.

## Introduction

Liver injury can be caused by hepatitis B virus, hepatitis C virus, hepatic lipidosis, aflatoxin B1, obesity, diabetes, and dietary habits [1, 2]. Hepatic lipidosis usually induces hepatic degeneration by excessive triglyceride accumulation, more than 80%–90% of hepatocytes, and impaired liver function. These factors damage hepatocytes and induce oxidative stress, resulting in the formation of free radicals such as reactive oxygen species (ROS) and reactive nitrogen species (RNS), which are produced by the oxidation-reduction cycle (redox) [3, 4]. These free radicals can induce hepatic stellate cells to generate collagen, leading to cirrhosis [5]. These free radicals can also induce lipid peroxidation and mitochondrial dysfunction in hepatocytes, and induce Kupffer cells to secrete cytokines, leading to immune cell infiltration and inflammation, leading to chronic hepatitis, cirrhosis, and the development of severe hepatocellular carcinoma (HCC) [6]. In addition, these free radicals can cause gene mutations or cause hepatocytes to release cytokines and chemokines that promote cell death and induce hepatocyte apoptosis, ultimately resulting in HCC [7–9]. Examination of liver disease can be performed by examining the clinical signs that may occur in the dog [10, 11]. Liver disease can be manifested in a wide variety of clinical signs depending on the vital function affected. The liver is the main organ involved in metabolism, detoxification, and storage function. Therefore, liver disease has a cascade effect on other body systems [10]. Clinical signs include depression, lack of appetite, vomiting, weight loss, diarrhea, polyuria, polydipsia, abdominal distention, lethargy, icterus, and ascites [12]. Neurological disorders are mainly caused by hypoglycemia and hepatic encephalopathy [13, 14]. Hepatic encephalopathy refers to a number of neurological symptoms that occur in dogs with liver disease, including seizures, disorientation, head pressing, blindness, or personality changes. It should be noted that most of the clinical signs are not specific to liver disease. Abdominal radiography, ultrasound examination, and liver biopsies tend to be complicated, inconvenient, and time-consuming methods for diagnosing liver disease. Liver disease can also be further diagnosed by laboratory results, including hematological features and serum biochemistry [13]. Hematologic features, including mild non-regenerative anemia, leukocytosis, and thrombocytosis, are commonly observed in canine liver tumors [15, 16]. Thrombocytosis is observed in approximately half of dogs with massive HCC [17]. Serum biochemistry indexes of dogs with liver disease commonly include elevated enzyme alanine transaminase, alkaline phosphatase, aspartate aminotransferase, hypoalbuminemia, hypoglycemia, hyperglobulinemia, and hyperbilirubinemia [18, 19].

However, most routine hematological and serum markers do not have high sensitivity or are not specific for liver disease diagnosis. Furthermore, the next step in the diagnosis of liver dysfunction is to identify novel markers for liver disease diagnosis [20]. Liver-fatty acid-binding protein (L-FABP) is highly expressed in the liver, kidney, lung, pancreas, and intestine [21, 22]. L-FABP is a key regulator of hepatic lipid metabolism that influences fatty acid uptake, transport, mitochondrial oxidation, and esterification [21]. In addition, L-FABP possesses cellular antioxidant properties because its antioxidant action is assumed to be mediated by methionine and cysteine amino acids that neutralize free radicals [23]. L-FABP binds many lipid peroxidation products and is present at high concentrations in hepatocytes [24]. Therefore, L-FABP may serve as an endogenous cellular protectant [25].

Previous research in humans has shown that serum L-FABP level is a prognostic factor and predictor of survival in patients with chronic liver disease, including chronic hepatitis, liver cirrhosis, and HCC [26]. Furthermore, another study showed that serum and urine L-FABP levels were strongly correlated with liver damage. Therefore, these biomarkers could be a non-invasive diagnostic marker for liver damage and could be used for follow-up [27, 28].

However, only a few studies of L-FABP in companion animals have been conducted, and the localization of L-FABP protein expression in dogs with liver injury has not been clearly investigated.

Therefore, the aim of this study was to investigate L-FABP expression in dogs with liver injury compared with that in dogs with normal liver function. This will provide further benefits for the development of L-FABP as a novel marker for liver injury diagnosis.

## Materials and Methods

### Ethical approval

The Animal Ethics Committee of the Faculty of Veterinary Medicine, Chiang Mai University, approved the use of all tissues and all methods (Ref. No. S18/2565).

### Study period and location

This study was conducted from January 2022 to January 2023. The present study was conducted at the Faculty of Veterinary Medicine, Chiang Mai University, Chiang Mai, Thailand.

### Collection and preparation of samples

Five liver tissue samples were analyzed (one from a dog with normal liver, one from a dog with lipidosis, and three from a dog with HCC). The tissue in the same area was selected and cut into blocks. All liver tissues were fixed in buffered 10% neutral formalin and prepared for water removal for embedding in paraffin. The automated tissue processor performed the biopsy samples at 37°C under alternating vacuum and pressure conditions for 12 h. Processed tissues were embedded into paraffin blocks with labeled labels and sectioned at 2 m on a rotary microtome. The tissue sections were stained with hematoxylin and eosin and prepared for immunohistochemistry to explore morphological changes and histochemical studies [29, 30].

### Immunohistochemistry

Liver sections were deparaffinized on a heat plate for 30 min. The liver sections were rehydrated with xylene for 20 min, followed by rehydration twice, 5 min each time. Absolute alcohol was rehydrated twice, 5 min each time; 95% alcohol was rehydrated twice, 3 min each time; 70% alcohol was rehydrated twice, 3 min each time; and dextrose 5% in distilled water (DW) washed for 2 min. The sections were boiled 5 times in antigen retrieval solution (1 mM Tris, pH 6.0) for 4 min and then left for 20 min at 25ºC. The sections were washed twice with DW for 2 min and twice with phosphate-buffered saline (PBS) for 5 min. Endogenous peroxidase was blocked with 33% H_2_O_2_ in methanol for 30 min and then washed twice with tap water for 2 min and PBS for 5 min. Non-specific binding was blocked with normal horse serum in PBS for 30 min in a moist chamber at 25ºC and then washed twice with PBS for 5 min. These sections were then incubated overnight in a moist chamber at 25ºC with purified anti-FABP1 antibody (Poly28601, Bio Legend, USA) diluted 1:400 in PBS with 0.05% NaNH_3_. The sections were rinsed twice in PBS for 5 min and then incubated for 1 h in a moist chamber at 25ºC with goat anti-rabbit immunoglobulin G H&L horseradish peroxidase secondary antibody (ab205718, Abcam, UK) diluted 1:800 in PBS for 1 h. The sections were rinsed with PBS twice, 10 min each time, and the antibody–antigen response sites were visualized using browning chromogen after 2 min of incubation with 0.05% 3,3′-diaminobenzidine tetrachloride (Dako, Denmark), which is dissolved in DW with 33% H_2_O_2_ and then rinsed in tap water for 2 min. Sections were counterstained with Mayer’s hematoxylin. After dehydration, the covers were mounted with a permanent mounting medium [31]. We collected and analyzed the results to compare the location and expression of L-FABP protein in dogs with normal liver and in dogs with liver injury, including lipidosis and HCC.

### Statistical analysis

The L-FABP staining results were analyzed using Image J software (http://rsb.info.nih.gov/ij/) available from the National Institutes of Health (Bethesda, MD), and the data were analyzed using inferential statistics (nonparametric statistics). Data are expressed as mean ± standard deviation (SD). Protein expression and localization between the three groups were compared using a one-way analysis of variance (ANOVA) using GraphPad Prism 9.0 (GraphPad Software, USA). Statistical significance was defined as p < 0.05 [31].

## Results

**Figure-1:**
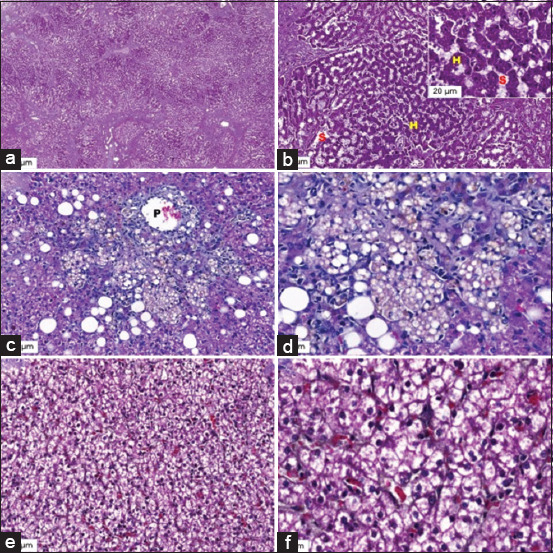
Low and high magnification (10×, 40×) of hematoxylin and eosin stained the section from (a and b) canine normal liver, (c and d) liver with lipidosis, and (e and f) liver with HCC. The normal liver showed normal histological structures without inflammatory cell infiltration. The liver with lipidosis showed the fatty degeneration of the hepatocytes at the periportal areas and inflammatory cell infiltration. The liver with HCC showed that the hepatocytes were polygonal-shaped with vacuolated cytoplasm, their nuclei were round to ovoid-shaped with stippled chromatin pattern and prominent nucleoli, the tumor cells were mild anisocytosis, anisokaryosis, and the tumor cells are arranged in solid pattern. H=Hepatocyte, S=Sinusoids, P=Portal vein, HCC=Hepatocellular carcinoma.

**Figure-2:**
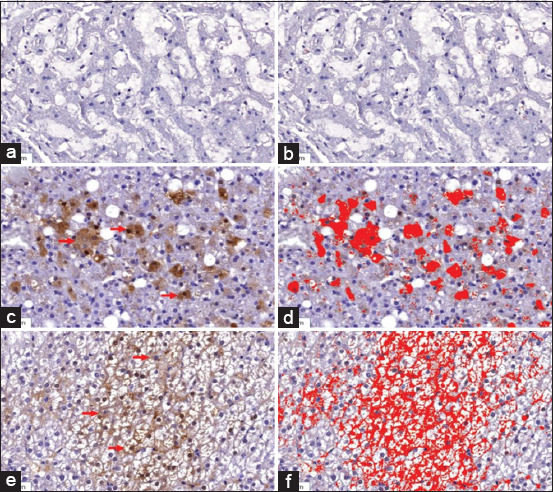
High magnification shows the immunohistochemical localization and immunolabeling of L-FABP in the sections of (a and b) normal liver, (c and d) liver with lipidosis, and (e and f) liver with HCC. The protein localization and expression were shown in brown color (red arrow). Immunohistochemical staining of L-FABP in the liver with lipidosis and liver with HCC revealed a strong expression of L-FABP in cytoplasm of hepatocyte (c and e). The immunolabeling revealed that L-FABP expression was lower in non-pathological liver (b) when compared to L-FABP expression in liver with lipidosis (d) and liver with HCC (f). L-FABP=Liver-fatty acid-binding protein, HCC=Hepatocellular carcinoma.

Histological liver sections with normal lesions (Figures-1a and b), lipidosis (Figures-1c and d), and HCC (Figures-1e and f) are shown in Figure-1. The histological structure of a normal liver showed normal structures without inflammatory cell infiltration (Figures-1a and b). A section of the liver with lipidosis revealed hepatocyte fatty degeneration at the periportal areas with mixed inflammatory cell infiltration (Figures-2c and d). Hepatocytes with anisocytosis, anisokaryosis, and tumor cells are arranged in a solid pattern (Figures-1e and f). Immunohistochemical localization of L-FABP in liver sections of normal lesions (Figures-2a and b), lipidosis (Figures-2c and d), and HCC (Figures-2e and f) are shown in Figure-2. The protein localization and expression are shown in brown. Immunohistochemical staining of L-FABP in the hepatocyte cytoplasm of the liver with lipidosis and liver with HCC revealed a strong expression of L-FABP (Figures-2c and e). In contrast, immunohistochemical staining of normal liver hepatocytes was less (Figure-2a). L-FABP expression was lower in the normal liver (Figure-2b) than in the liver with lipidosis (Figure-2d) and the liver with HCC (Figure-2f).

**Figure-3:**
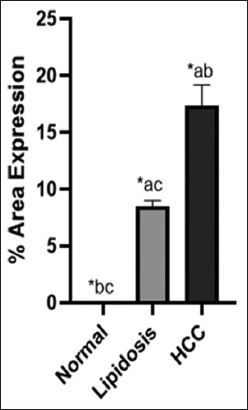
Semi-quantitative analysis of liver-fatty acid-binding protein in normal liver, liver with lipidosis and liver with HCC. The one-way ANOVA values are expressed as mean and standard deviation, with p < 0.05* significance level (a; compared to normal, b; compared to lipidosis and c; compared to HCC). HCC=Hepatocellular carcinoma.

Semiquantitative analysis of protein expression and location within liver tissues using immunohistochemistry data is presented as the mean ± SD of the mean to represent protein expression and localization between non-pathological and liver injury, including liver with lipidosis and liver with HCC (Table-1). The percentage of protein expression of L-FABP in the non-pathological liver was 0.023 ± 0.027, whereas that in the liver with lipidosis and HCC was 8.517 ± 1.059 and 17.371 ± 4.026, respectively. Moreover, a statistical comparison of L-FABP expression and localization using one-way ANOVA in Figure-3 (p = 0.05) showed that the percentage of L-FABP expression was significantly increased in liver injury, including liver with lipidosis and liver with HCC, compared with that in the non-pathological liver. Our study showed that the liver with HCC significantly increased the expression of L-FABP compared to the liver with lipidosis.

**Table 1:** The semi-quantitative evaluation of protein L-FABP expression (percentage area) as the mean ± SD on normal liver, liver with lipidosis and liver with HCC. The one-way ANOVA values are given as mean ± SD (p < 0.05).

Protein expression (%area)	L.FABP
Normal liver	0.023 ± 0.027
Lipidosis	8.517 ± 1.059*
Hepatocellular carcinoma	17.371 ± 4.026*

Mean ± standard deviation (SD): Significantly different compared to non-pathological liver (p < 0.05)*, HCC=Hepatocellular carcinoma, L-FABP=Liver acid binging protein, ANOVA=Analysis of variance

## Discussion

The present study elucidated the expression of L-FABP in dogs with liver injury compared with that in dogs with normal liver. L-FABP was significantly increased in dogs with liver injury, including lipidosis and HCC. Interestingly, L-FABP is strongly correlated with liver damage through oxidative stress mechanisms. It has been demonstrated that L-FABP plays an important role as an efficient endogenous antioxidant and cytoprotective in the liver. L-FABP may control the availability of long-chain fatty acids (LCFAs) and their oxidative metabolites with ligands. Resulting in limiting the free LCFA fraction, modulating the interaction of LCFAs with nuclear receptors, and trapping or scavenging reactive species such as ROS and RNS are known to be effective antioxidant agents participating in S-thiolation/de-thiolation reactions, and several methionine groups [32, 33]. Methionine residues are considered cellular scavengers of activated xenobiotics such as carcinogens [25]. L-FABP regulates fatty acid metabolism through an association with the peroxisome proliferator-activated receptor- alpha (PRAR-a) in beta-oxidation and is involved in hepatocellular damage through oxidative stress mechanisms [26]. In a previous study [34], oxidative stress was stimulated by alcohol use as a predisposing factor in wild-type mice with L-FABP genes and in mice lacking the L-FABP gene (L-FABP/mice) fed a modified Lieber–DeCarli liquid (ethanol) diet for 6 weeks. L-FABP/mice displayed prominent oxidative protein expression in liver sections of ethanol-fed mice. Moreover, elevated glutathione, thiobarbituric acid reactive substance, 8-isoprostanes, and carbonyl content of proteins indicated that L-FABP/mice exhibited high sustained oxidative stress in the liver. L-FABP is an indirect antioxidant protein essential for sequestering free fatty acids and decreasing the pathogenesis of alcoholic liver disease [34, 35].

Furthermore, L-FABP expression was higher in HCC compared with lipidosis. The results indicated that HCC is more severe and induces oxidative stress. This finding is consistent with other previous studies by Ku *et al*. [27], including the study of L-FABP expression in patients with HCC, which showed that L-FABP was highly expressed in HCC tissues compared with normal or degenerative liver tissues [26]. Another immunohistochemical study of L-FABP expression in 23 childhood hepatoblastomas and 62 adult HCCs revealed that all hepatoblastomas and half of the HCCs contained L-FABP-immunoreactive tumor cells [36]. Lower L-FABP expression is related to reduced fatty acid accumulation and uptake in mice [37]. The results of this study suggest that the degree of fatty acid metabolism may variably change L-FABP expression, especially in hepatic lipidosis. Compared with HCC, fatty acid metabolism continuously increases according to tumor cell severity [37].

At present, plasma L-FABP is used as a novel biomarker for injury in many organs, especially the kidneys and intestines, which are the main sources of L-FABP production. The study of L-FABP used as a biomarker of acute kidney injury (AKI) showed that urinary L-FABP elevation correlates with the severity of AKI, particularly in the early stages, and can be used for drug administration or tissue transplant monitoring [38, 39]. Serum L-FABP and trefoil factor-3 levels decrease in the case of displaced abomasum (DA) in ruminants in contrast with increasing leptin levels. This alteration of protein pattern may suggest impaired blood perfusion due to DA, gastrointestinal epithelial damage, and liver lipidosis [40]. Moreover, L-FABP plasma concentration was found to increase in dogs with inflammatory bowel disease [41]. Furthermore, another study of the association between L-FABP protein and vascular endothelial growth factor A (VEGF-A) in the tissues of patients with HCC showed a positive correlation between L-FABP expression and VEGF-A expression [27]. In addition, L-FABP significantly induces VEGF-A upregulation and increases angiogenic potential and migration activity in HCC cells by activating VEGF receptor-2 on membrane rafts and subsequently activating the Akt/mTOR/P70S6K/4EBP1 and Src/FAK/cdc42 pathways, suggesting that L-FABP may be a potential therapeutic target in HCC therapy [21, 42]. According to the limited studies on liver and gastrointestinal injury markers, L-FABP may be an interesting marker of liver and gastrointestinal injury in both humans and animals.

## Conclusion

This was a preliminary study of L-FABP levels in dogs with liver injury. A significant increase in L-FABP expression was observed in dogs with liver injury due to antioxidant ability. Our findings can be instigated with other previous studies and used as a database for further development of liver injury markers. L-FABP may, therefore, be elevated in the serum or plasma of dogs with liver injury. Plasma LFABP levels may be useful as a diagnostic and prognostic tool for dogs with liver injury and associated signs.

## Authors’ Contributions

PM, JA, NC, and NS: Study concept and conducted the study. NC and NS: Collected the sample and drafted the manuscript. PM and JA: Formal analysis and data curation, provided scientific advice, review, and editing. All authors have read, reviewed, and approved the final manuscript.
